# Quantitative Comparative Analysis of Annual Training Volume and Intensity Distribution of Male Biathlon National Team and University Athletes Using Global Positioning Systems and Wearable Devices

**DOI:** 10.3390/s26061910

**Published:** 2026-03-18

**Authors:** Guanmin Zhang, Qiuju Hu, Yonghwan Kim, Yongchul Choi

**Affiliations:** 1 Department of Physical Education, Gangneung-Wonju National University, Gangneung 25457, Republic of Korea; zhangguanmin@gwnu.ac.kr (G.Z.); 20258071@gwnu.ac.kr (Q.H.); 2 Laboratory of Integrated Physiology, Department of Health and Human Performance, University of Houston, Houston, TX 77204, USA

**Keywords:** biathlon, training volume, training intensity distribution, specificity, long-term athlete development, GPS monitoring, wearable sensors

## Abstract

Background: Wearable sensors and global positioning systems (GPS) can enable objective monitoring of training loads in outdoor endurance sports. In biathlons, comparing training characteristics across developmental stages can help identify structural gaps and support evidence-informed progression within long-term athlete development (LTAD). This study aimed to quantitatively compare the annual training characteristics of Korean male biathlon national team (NT) and university (UNV) athletes. Methods: Annual physical training data (2022–2024) from NT (n = 6) and UNV (n = 6) athletes were collected using Catapult Vector S7 GPS devices and Polar H10 heart rate monitors. Training volume, intensity distribution (zones 1–3 based on %HRmax), modality (skiing vs. running), and periodization were compared using Mann–Whitney U tests with rank-biserial correlation (r_rb). Results: NT athletes accumulated a higher annual training time and distance than UNV athletes (812 vs. 606 h; 6359 vs. 4130 km; *p* = 0.002, r_rb = 1.000 for both). The NT athletes spent a lower proportion of time on low-intensity training and a higher proportion on mid and high intensities than UNV athletes (*p* ≤ 0.015). During high-intensity training, NT athletes maintained a higher proportion of ski-specific training, whereas UNV athletes relied more on running (skiing: 78.5% vs. 46.4%; running: 21.5% vs. 53.6%; both *p* < 0.001, r_rb = 1.000). The UNV group also showed a more concentrated structure during competition periods than NT athletes (COMP: 28.3% vs. 14.6%; *p* < 0.05). The absolute annual strength training time did not differ, but UNV athletes showed a higher strength ratio (23.3% vs. 16.8%; *p* < 0.001, r_rb = 1.000). Conclusion: UNV athletes exhibited a lower total volume, more low-intensity-skewed distribution, and reduced ski-specific exposure during high-intensity training compared with NT athletes. These observed structural gaps can provide empirical benchmarks that may help coaches plan stage-appropriate progression, and they illustrate the practical value of GPS- and wearable-based monitoring for identifying training divergences across developmental stages.

## 1. Introduction

In recent years, wearable sensors and global positioning systems (GPS) have become increasingly valuable tools for objective, continuous monitoring of athlete training loads in outdoor sports [1,2]. These technologies enable real-time acquisition of key performance parameters, including movement speed, distance covered, and heart-rate-derived exercise intensity, without disrupting the natural training environment. In endurance sports such as biathlons, where athletes train across vast outdoor terrains, GPS-based monitoring provides a particularly effective means of capturing spatial movement patterns and quantifying training volume and intensity over extended periods [3]. This sensor-based approach offers significant advantages over traditional subjective training logs by providing objective, reproducible data that can inform evidence-based training program designs.

A biathlon combines cross-country skiing with precision rifle shooting, demanding high aerobic power, efficient skiing techniques, and the ability to stabilize and shoot accurately under physiological stress. Wearable sensors and GPS can capture key external and internal load indicators in this context, such as the distance and speed across terrain together with heart-rate-derived intensity responses [4,5]. Accordingly, performance is influenced by annual training volume, training intensity distribution (TID), sport-specific modality use, and long-term periodization [6,7].

Previous research has established core principles of endurance training relevant to skiing-based sports, including frameworks for TID [6] and descriptions of elite cross-country skiing training routines [8,9]. Recent biathlon-specific work has also provided detailed case study insights into long-term development and elite training structure [10,11,12,13]. Collectively, these studies suggest that world-class endurance athletes typically accumulate high annual training volumes with a predominance of low-intensity work, while systematically integrating moderate- and high-intensity sessions and increasing sport specificity as the competition season approaches [11,14,15].

In Korea, research on winter endurance sports has focused mainly on cross-country skiing, including physiological and technical analyses and training design studies [16,17]. However, biathlons add unique constraints—most notably the requirement to maintain shooting accuracy after intense skiing—so direct transfer of cross-country skiing evidence is limited. Biathlon-specific evidence is therefore needed to better understand training demands and to support athlete development in this context [4,7].

Within the long-term athlete development (LTAD) framework, understanding structural differences in training across developmental stages is essential for designing evidence-informed progression pathways. Comparing athletes across stages (e.g., national team versus university level) extends beyond documenting the expected differences in total training volumes. Such comparisons can reveal qualitative shifts in training structures—such as changes in intensity distribution, sport-specific training emphasis, and periodization strategies—that are likely required for the successful progression to elite performance [10,18].

Identifying these structural gaps can provide actionable information for coaches and practitioners. For example, detecting a reliance on general running rather than ski-specific modalities during high-intensity training at the university stage suggests a need to progressively introduce sport-specific stimuli and balance periodization across the preparatory phases. This may help reduce developmental stagnation and support a smoother transition to elite-level demands. In contexts where the biathlon athlete pool is extremely limited, such as in Korea, optimizing the quality of training progression at each developmental stage is particularly important for long-term competitiveness.

The objective of this study is to quantitatively compare the annual training volumes, intensity distributions, training-type compositions, and periodization patterns between Korean male biathlon national team (NT) and university (UNV) athletes using wearable sensors and GPS. By identifying qualitative structural gaps, this study aims to provide empirical benchmarks that can inform the design of progressive long-term training pathways.

## 2. Methods

### 2.1. Research Design and Participants

This study utilized an observational comparative design to examine annual training characteristics of Korean male biathlon athletes across developmental stages. Training variables were quantified via objective sensor-based monitoring without experimental manipulation. The study period was 2022–2024, and the analysis examined the total annual training volumes, training intensity distributions by zone, zone × type combinations, periodized distributions across phases, and the composition of strength training.

A total of 12 elite South Korean male biathlon athletes participated, including a national team group (NT; n = 6) and a university group (UNV; n = 6) affiliated with the Korea Biathlon Federation. Given the size of the Korean biathlon system, this cohort represented a near-census of athletes competing at each level. Training intensity distribution (TID) was categorized using the three-zone model proposed by Seiler et al. [6]. Core athletes were monitored across the three seasons (2022–2024), and values were averaged across seasons to represent annual characteristics; minor roster changes related to team selection did not alter the overall composition of each group.

This study focused on the physical load underlying biathlon performance. Therefore, stationary shooting drills and trigger practices without meaningful increases in heart rate or movement were excluded. Only physical training sessions such as skiing, running, and strength training were included.

### 2.2. Data Processing and Quantitative Analysis

This study used training time and distance as indicators of training volume to quantitatively evaluate annual training. All training data were collected through annual training measurements for each athlete, and each training session included the training type, training time, training distance, training intensity (Zones 1 to 3), and training period (months). This study focused on analyzing the intensity zones using distance and heart rate; specifically, training intensity zones were classified exclusively based on heart rate (%HRmax) using the three-zone model, while distance was used as a complementary indicator of training volume but not for intensity zone classification.

**Figure 1:**
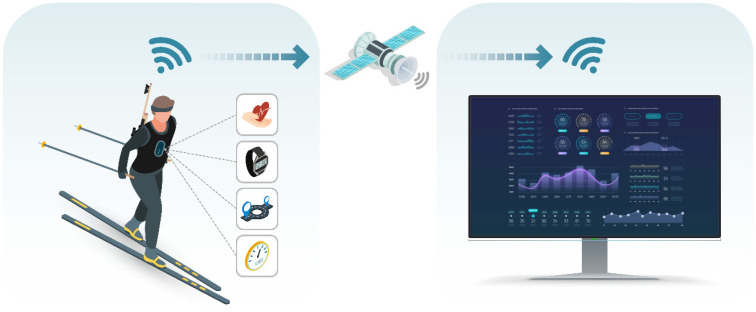
Data acquisition process with global positioning system and wearable sensor device.

Training volume distance analysis was performed using a wearable Vector S7 device (Catapult Innovations, Melbourne, VIC, Australia) capable of GPS monitoring, and the collected data were analyzed using Catapult software (OpenField, version 1.22.2, Catapult Group Holdings Limited, Melbourne, VIC, Australia) (Figure 1). The device demonstrated high reliability in inertial motion analysis, which measured the accuracy of movements such as acceleration, deceleration, changes in direction, and jumping. The intraclass correlation coefficient was 0.97–1.00, which indicated that it was an appropriate tool for measuring physical activity levels [3]. The heart rate was measured using Polar H10 heart rate monitors (Polar Company, Kempele, Finland), and data were analyzed using Polar Flow software 4.0.6 (Polar Company, Kempele, Finland) [19]. Heart rate was monitored using a wrist-worn watch system and simultaneously transmitted to a computer. The training volume was recorded daily through a training log, and the collected data included the training time, distance, exercise type, and heart rate.

The maximal heart rate (HRmax) was determined using a graded exercise test (GXT) conducted at the beginning of each training year under standardized laboratory conditions. The relative intensity (%HRmax) was calculated for each athlete based on the individually measured HRmax. The three-zone model proposed by Seiler (2010) was applied for training the intensity classification [6]. Accordingly, a heart rate below 82% of the maximum heart rate was classified as Zone 1 (LOW), 82–87% as Zone 2 (MID), and >87% as Zone 3 (HIGH). Fixed %HRmax thresholds were adopted based on Seiler’s three-zone model, which provided a practical and widely validated framework for field-based intensity monitoring in endurance sports. It should be noted that heart-rate-based intensity classification has inherent limitations, including cardiac drift during prolonged exercise, the effects of ambient temperature and altitude, and individual variability in the heart-rate-intensity relationship. These limitations are discussed in Section 4.

**Figure 2:**
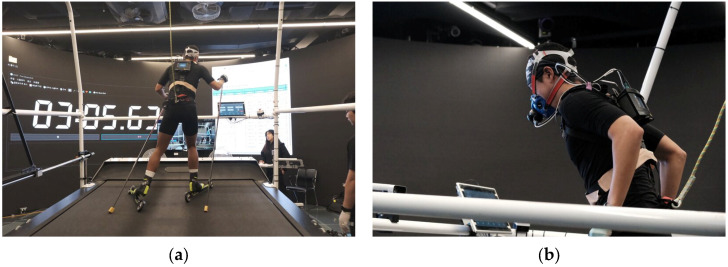
Graded exercise test (GXT) on a roller ski treadmill with respiratory gas analysis using the COSMED K5 Wearable Metabolic System. (**a**) Rear view of the athlete performing the GXT on the roller ski treadmill with the portable metabolic analyzer; (**b**) close-up view showing the COSMED K5 face mask and wearable unit during the incremental test protocol.

Specifically, GXT was performed on a roller ski treadmill under controlled laboratory conditions. During the test, respiratory gas exchange was continuously measured using the COSMED K5 Wearable Metabolic System (C09090-01-99, COSMED, Rome, Italy), which is a portable breath-by-breath metabolic analyzer that allows real-time measurement of VO_2_, VCO_2_, ventilation, and respiratory exchange ratios during sport-specific exercise. The use of a roller ski treadmill enabled the GXT to closely replicate the biomechanical demands of on-snow skiing, thereby enhancing the ecological validity of HRmax and ventilatory threshold measurements. When feasible, GXT was repeated during the annual training cycle to verify HRmax values and to adjust individual training intensity zones for seasonal changes in cardiovascular fitness, helping to maintain accurate intensity zone classification throughout each training year (Figure 2). The training distance was measured using GPS, and all distance data were standardized to kilometers (km). The training time, recorded in minutes, was converted to hours for analysis.

### 2.3. Data Curation

The total annual training volume was calculated as the cumulative sum of each athlete’s annual training time and distance, and the mean and standard deviation were calculated for both NT and UNV groups. To analyze the training volume by intensity, each training session’s total time was partitioned into low, mid, and high zones based on the time spent in each heart-rate zone within that session. For sessions containing multiple intensity phases (e.g., warm-up in Zone 1, followed by high-intensity intervals in Zone 3), the within-session time was proportionally allocated to the respective zones. The training time and distance for each zone were summed, and the percentages of each zone relative to the total annual training time and distance were calculated.

To analyze the relationship between training intensity and type, a zone × type analysis was performed. Within each zone, the training time for each type was summed and normalized to 100% of the total training time for that zone to analyze the characteristics of the composition type within each intensity range.

For periodic training volume analysis, annual training was divided into monthly periods and then reclassified into General Preparation Period 1 (G1, April to June), General Preparation Period 2 (G2, July to September), Specific Preparation Period (SP, October to November), and Competition Period (COMP, December to February). The training times for each period was summed, and the percentages relative to the total annual training time were calculated to analyze periodization characteristics.

In addition, for strength training analysis, dedicated sessions consisting primarily of weight training, core training, functional training, and small-equipment training were classified as strength training. For mixed sessions that combined endurance and strength components, the time was allocated to each training category based on the actual time spent on each component, as recorded in the daily training logs, ensuring that strength training time reflected only the actual strength-focused portion of each session. The annual training time for strength training and the percentage of the total training time were calculated to compare the differences between the NT and UNV groups.

### 2.4. Data Analysis

Statistical analysis of the collected data was performed using SPSS 27.0 (IBM Corp., Armonk, NY, USA). Descriptive statistics, including mean and standard deviation, were calculated for all dependent variables. The Mann–Whitney U test, a non-parametric test method, was used to compare the training volumes between groups. The Mann–Whitney U test was selected because it does not assume a normal distribution and is appropriate for small samples. The effect sizes were calculated using rank-biserial correlation. To interpret the power, we calculated sensitivity using G*Power 3.1.9.7 (Universität Düsseldorf, Düsseldorf, Germany). This study confirmed its power through sensitivity analysis. High power was considered to be 0.80, and the calculation conditions were as follows: Mann–Whitney U test; group 1 = 6 and group 2 = 6; α = 0.05. The result was an effect size of d = 1.5, which, when converted to the rank-biserial correlation used in this study, yielded a minimum detectable effect size of r = 0.75. This can be interpreted as a threshold; therefore, a value of 0.75 or higher was considered high power, and a value of 0.75 or lower was considered low power. Consequently, the lower the power, the greater the likelihood of Type II errors. All statistical significance levels were set at *p* < 0.05.

## 3. Results

### 3.1. General Characteristics

The general characteristics of the participants are listed in Table 1. The NT group was significantly older and had lower heights and weights than the UNV group (age, height, weight: *p* < 0.001, r_rb = 1.000). VO_2_ peak and ventilation threshold, which are indicators of cardiorespiratory fitness capacity, were significantly higher in the NT group. However, there were no significant differences in BMI or body fat percentage between the two groups (*p* > 0.05).

### 3.2. Annual Training Volume and the Time and Distance of Training

Table 2 compares annual training volumes between the NT and UNV groups. NT athletes showed significantly higher total annual training time and distance than UNV athletes (both variables: *p* = 0.002, r_rb = 1.000). Regarding distance, the UNV group’s training was disproportionately concentrated in the low-intensity zone (*p* = 0.002, r_rb = 1.000), whereas NT athletes allocated a significantly larger proportion of training to high intensity when compared with UNV athletes (*p* = 0.015, r_rb = 0.833).

### 3.3. Training Intensity and Type

Training time ratios by zone × type are presented in Table 3. There was no significant difference between groups in the relative contribution of skiing and running within the low-intensity zone (both variables: *p* = 0.310, r_rb = 0.389). In the high-intensity domain, NT athletes had a higher proportion of ski training, while UNV athletes relied more on running training (both variables: *p* < 0.001, r_rb = 1.000).

### 3.4. Training Time and Ratio Based on Periodization

**Figure 3:**
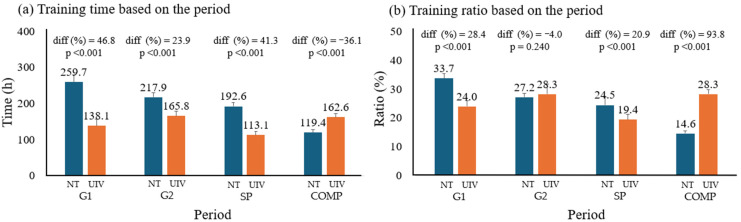
Training time and ratio based on periodization; (**a**) training time based on the period; (**b**) training ratio based on the period; *p* < 0.05; NT, national team; UNV, university; diff, difference; G1, General Preparation Period 1 (April to June); G2, General Preparation Period 2 (July to September); SP, Specific Preparation Period (October to November); COMP, Competition Period (December to February).

Figure 3 shows the distribution of training times by periodization phase. In training ratio, the NT group exhibited a structure centered on the preparatory phase (G1, and SP) (both variables: *p* < 0.001), while the UNV group exhibited a structure centered on the competitive period (COMP) (*p* < 0.001).

### 3.5. Strength Training Volume

Table 4 summarizes the annual composition of strength training. Absolute annual strength training time did not differ significantly between groups (*p* < 0.001, r_rb = 1.000); however, the relative proportion of strength training was higher in UNV athletes due to their lower total endurance training time (*p* < 0.001, r_rb = 1.000).

## 4. Discussion

This study used wearable sensors and GPS to compare annual training structures between Korean male biathlon national team (NT) and university (UNV) athletes. By combining external load (time, distance) and internal load (heart-rate-derived intensity zones), the analysis aimed to identify structural differences that may inform stage-appropriate progression within an LTAD perspective.

NT athletes accumulated higher annual training exposure and allocated a greater share of training to mid and high intensity than UNV athletes. While high-volume, low-intensity training has been a hallmark of elite endurance preparation, LTAD-oriented comparisons can be particularly informative when they reveal how higher-intensity stimuli can be integrated across stages. In biathlons, these stimuli are closely linked to competitive pace tolerance and the ability to maintain technical quality under fatigue. Longitudinal evidence from elite athletes can provide informative benchmarks, such as a 17-year case study which documented training volumes increasing from approximately 522 h/year at age 20 to 937 h/year during peak performance [12], suggesting that UNV athlete volumes (~605 h) may represent a developmentally appropriate starting point. The 80/20 distribution principle derived from Olympic medalists [15] could offer a reference against which both NT (87.2% low intensity) and UNV (95.1%) athletes deviate—with the UNV group’s deviation being substantially larger. Sylta et al. [20] further showed that the strategic allocation of high-intensity training zones was more decisive for endurance adaptations than simply accumulating low-intensity volume.

A key qualitative gap was observed in the modality used during higher-intensity work. The NT athletes maintained predominantly ski-based training in the mid and high zones, whereas the UNV athletes relied more on running at high intensity. Running can support general aerobic development, but ski-specific high-intensity exposure was more likely to provide specific neuromuscular and technical stimuli relevant to biathlon skiing. Therefore, the observed modality shift at the university stage may represent an important target for progressive integration of sport-specific high-intensity sessions. This interpretation is supported by Sandbakk et al. [21], who showed that cross-country skiers performing high-intensity sessions with sport-specific modalities achieved superior physiological adaptations compared with those relying on running-based training. Similarly, the transition from junior to senior world-class level has been characterized by a marked increase in ski-specific high-intensity session frequency [18], reinforcing the developmental importance of this modality shift.

Periodization patterns further differentiated the groups: the NT group distributed training more evenly across preparatory phases, whereas the UNV group concentrated a larger share of annual training during the competition period. A preparation-dominant structure was generally consistent with building aerobic and technical foundations before increasing specificity and competition demands, whereas a competition-period concentration may reflect logistical constraints but could limit systematic progression. Longitudinal case study evidence has supported this interpretation, showing that world-class athletes systematically increased high-intensity session frequency from general preparation toward competition phases rather than concentrating training load during the competition period alone [12,13].

Absolute strength training time was similar between the two groups, and no significant differences were observed. However, the UNV group had a higher proportion of strength training because they had less endurance training. This suggests that the key issue is not simply adding strength work but balancing the endurance–strength composition to support sport-specific development across the season.

Considering the small number of participants, this study conducted a sensitivity analysis to assess the power of the test. Lower power increases the likelihood of a Type II error. This study calculated power using effect sizes. Although this study may not find significant differences, it is important to consider the increased risk of a Type II error if the number of participants is small, the significance level is too low, or the actual difference is small.

From a methodological perspective, this study demonstrated the utility of integrating GPS and wearable heart rate monitors for long-term training surveillance in biathlons, enabling the objective identification of where training structures diverge across developmental stages.

This study has several limitations. First, the sample size (n = 6 per group) limits generalizability, although it represented a near-census of elite Korean male biathlon athletes at each level. Many of the primary comparisons in this study yielded statistically significant results despite the small sample size, and the associated effect sizes were consistently large (rank-biserial correlations often exceeding 0.90). This suggested that the observed differences in key variables—such as total annual training volume, training intensity distribution, and the proportion of sport-specific high-intensity training—were sufficiently strong that insufficient statistical power was unlikely to have substantially influenced these findings. Therefore, the small sample size did not appear to have critically limited the interpretation of the main significant results. However, the possibility of Type II errors should be considered when interpreting results that were not statistically significant. For example, the comparison of absolute strength training time between the NT and UNV groups showed no significant difference (*p* > 0.05). Given the limited statistical power to detect smaller effects, it is possible that a meaningful difference existed but was not detected in the present sample. Therefore, this result should not be interpreted as definitive evidence that the two groups perform identical amounts of strength training, but rather as an observation that requires further investigation with larger samples. Overall, the small sample size primarily limited the ability of this study to detect smaller effects rather than the validity of the large differences observed in several key training variables. Future studies including larger samples, additional teams, and longitudinal datasets will be necessary to confirm these findings and further refine the understanding of training structure differences across developmental stages in biathlon athletes. Second, group differences should be interpreted as empirical benchmarks rather than prescriptive guidelines, because optimal training structures may vary by individual needs and constraints. Third, heart-rate-based intensity classification has known limitations (e.g., cardiac drift and environmental influences). Future work should include larger cohorts (including female and youth athletes) and integrate shooting performance, physiological indicators, and competition outcomes to refine evidence-informed biathlon development models.

## 5. Conclusions

This study quantitatively compared training structures of national team (NT) and university (UNV) biathlon athletes using wearable sensors and GPS. The NT group exhibited a more balanced annual structure, characterized by a higher total volume, a greater proportion of mid- and high-intensity training, a predominant use of ski-specific modalities during high-intensity sessions, and a preparation-focused periodization. In contrast, UNV athletes showed a lower total exposure, an intensity distribution more skewed toward low intensity, a greater reliance on running during high-intensity work, and a more competition-period-concentrated training distribution. Together, these stage-related differences—particularly the reduced ski-specific high-intensity exposure in UNV athletes—have highlighted concrete developmental gaps that can be monitored and addressed through structured progression planning. The GPS- and wearable-based approaches used here can provide a practical foundation for evidence-informed training evaluations and long-term athlete development in biathlons.

## Figures and Tables

**Table 1:** General characteristics of the study participants.

Variables	NT (n = 6)	UNV (n = 6)	r_rb	*p* Value
Age, years	28.0 ± 1.4	24.5 ± 0.7	1.000	<0.001
Height, cm	167.5 ± 0.7	173.9± 5.5	1.000	<0.001
Weight, kg	59.5 ± 2.1	65.0 ± 2.9	1.000	<0.001
BMI, kg/m^2^	21.2 ± 0.9	21.5 ± 1.7	0.001	0.891
Fat, %	11.5 ± 2.1	10.8 ± 0.4	0.222	0.584
VO_2_ peak, mL/kg/min	79.3 ± 1.5	75.2 ± 2.4	1.000	<0.001
Ventilatory threshold, % VO_2_max	78.0 ± 1.4	74.5 ± 2.1	1.000	<0.001
Athletic experience, years	11.4 ± 3.3	6.9 ± 1.9	1.000	<0.001

*p* < 0.05; NT, national team; UNV, university; r(rb), rank-biserial correlation; BMI, body mass index.

**Table 2:** Annual training volume and the ratio of training time and distance by training intensity.

Variables	NT	UNV	Diff (%)	r_rb	*p* Value
Annual training time (h)	812.2 ± 39.0	605.6 ± 34.4	25.4	1.000	0.002
Annual training distance (km)	6358.7 ± 227.1	4129.8 ± 169.8	35.1	1.000	0.002
Time (%)					
LOW (Zone 1)	87.2 ± 1.8	95.1 ± 1.4	−9.0	1.000	0.003
MID (Zone 2)	6.0 ± 0.7	2.6 ± 0.3	56.7	0.944	0.004
HIGH (Zone 3)	6.8 ± 0.9	2.3 ± 0.4	66.2	0.944	0.004
Distance (%)					
LOW (Zone 1)	86.4 ± 2.1	92.0 ± 1.6	−6.5	1.000	0.002
MID (Zone 2)	6.1 ± 0.7	4.5 ± 0.4	26.2	1.000	0.002
HIGH (Zone 3)	7.5 ± 1.5	3.5 ± 0.7	53.3	0.833	0.015

*p* < 0.05; NT, national team; UNV, university; diff, difference; r(rb), rank-biserial correlation.

**Table 3:** Training time ratios based on training intensity and type.

Zone	Type	NT (%)	UNV (%)	Diff (%)	r_rb	*p* Value
LOW	skiing	57.4 ± 3.3	58.1 ± 4.1	−1.2	0.389	0.310
	running	42.6 ± 2.3	41.9 ± 3.1	1.6	0.389	0.310
MID	skiing	84.6 ± 5.4	78.4 ± 5.6	7.3	1.000	0.002
	running	15.4 ± 1.4	21.6 ± 3.6	−40.2	1.000	0.002
HIGH	skiing	78.5 ± 5.2	46.4 ± 2.3	40.9	1.000	<0.001
	running	21.5 ± 2.2	53.6 ± 2.9	−149.3	1.000	<0.001

*p* < 0.05; NT, national team; UNV, university; diff, difference; r(rb), rank-biserial correlation.

**Table 4:** Annual strength training time and ratio.

Variables	NT	UNV	Diff (%)	r_rb	*p* Value
Absolute strength training time (h)	135.3 ± 11.8	131.3 ± 12.2	3.0	0.444	0.180
Total endurance training time (h)	803.4 ± 32.2	562.6 ± 27.1	30.0	1.000	<0.001
Strength ratio (%)	16.8 ± 1.7	23.3 ± 2.4	−38.6	1.000	<0.001

*p* < 0.05; NT: national team; UNV: university; diff: difference; r(rb), rank-biserial correlation. Note: Total training time in Table 4 reflects the sum of endurance and strength training sessions only, excluding supplementary activities (e.g., shooting drills and recovery sessions) not categorized as endurance or strength training. This accounts for the difference in total annual training time, as reported in Table 2.

## Data Availability

The data presented in this study are available on reasonable request from the corresponding author.
